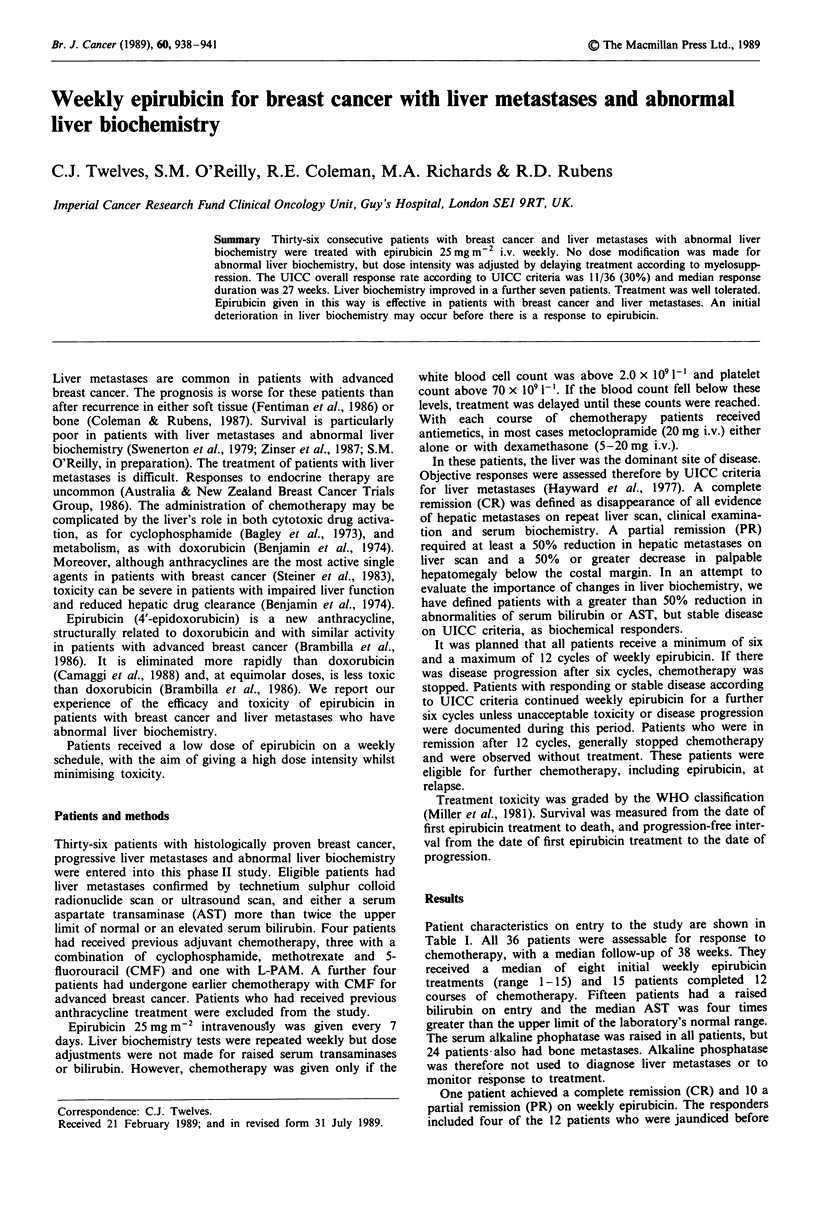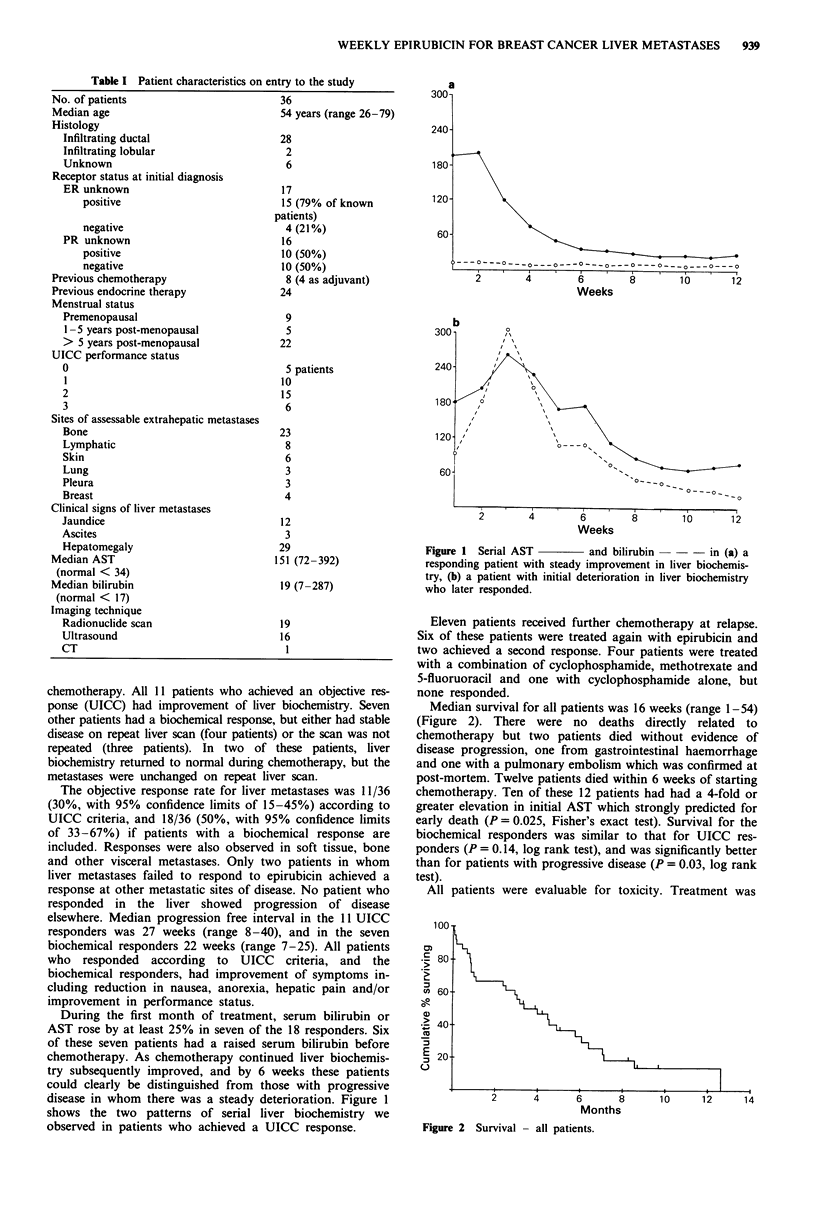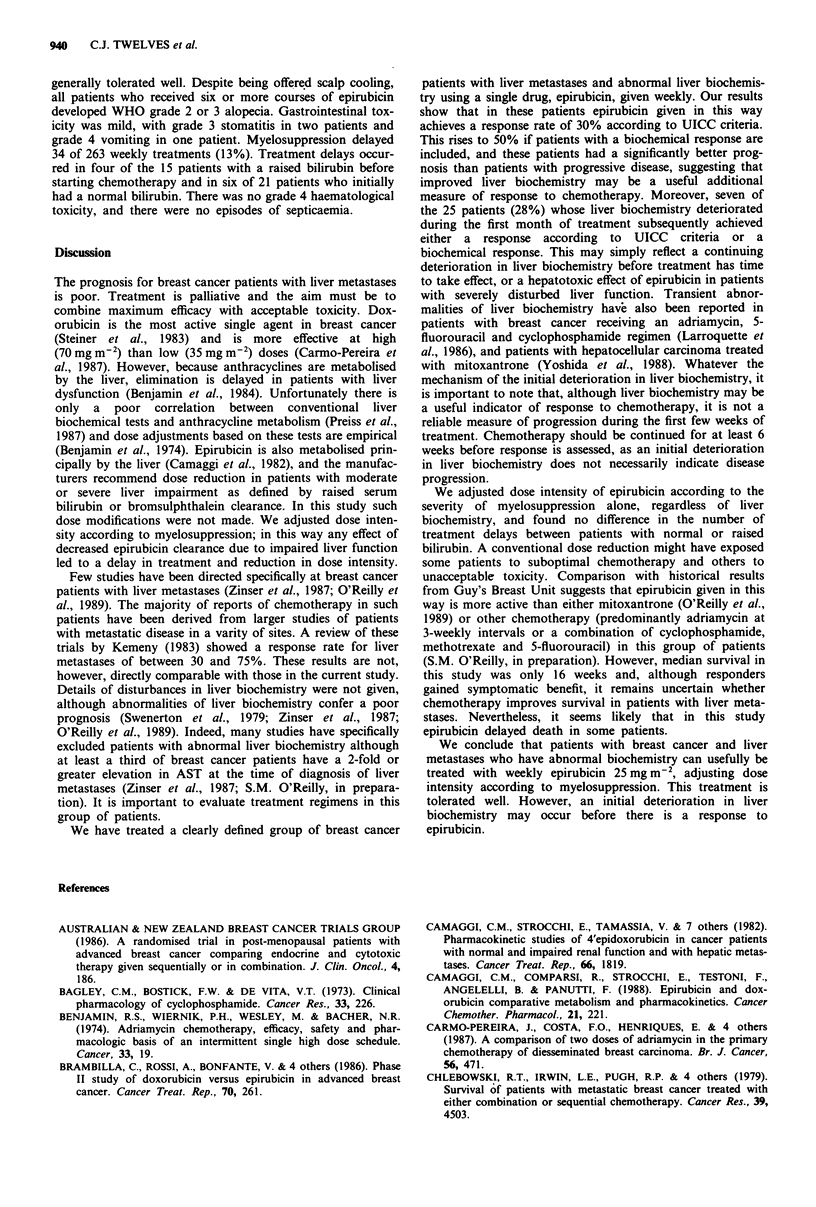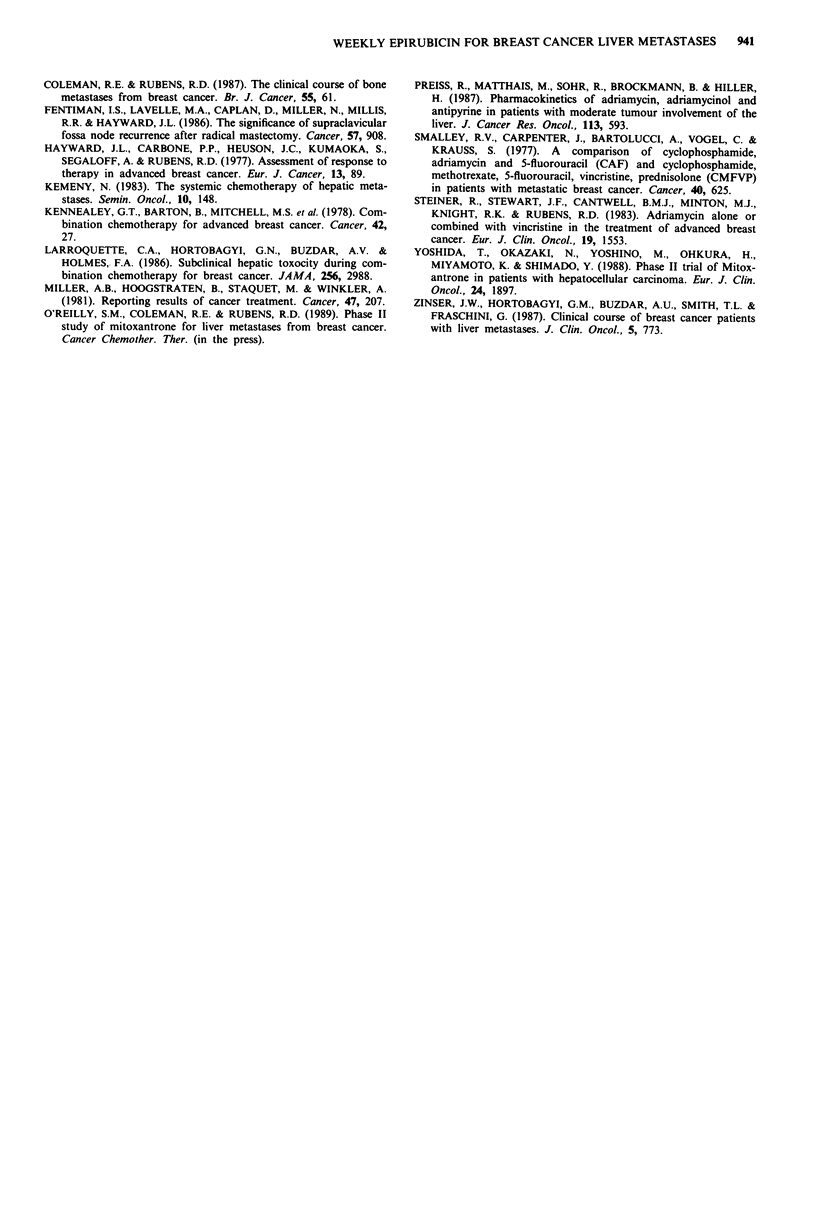# Weekly epirubicin for breast cancer with liver metastases and abnormal liver biochemistry.

**DOI:** 10.1038/bjc.1989.394

**Published:** 1989-12

**Authors:** C. J. Twelves, S. M. O'Reilly, R. E. Coleman, M. A. Richards, R. D. Rubens

**Affiliations:** Imperial Cancer Research Fund Clinical Oncology Unit, Guy's Hospital, London, UK.

## Abstract

Thirty-six consecutive patients with breast cancer and liver metastases with abnormal liver biochemistry were treated with epirubicin 25 mg m-2 i.v. weekly. No dose modification was made for abnormal liver biochemistry, but dose intensity was adjusted by delaying treatment according to myelosuppression. The UICC overall response rate according to UICC criteria was 11/36 (30%) and median response duration was 27 weeks. Liver biochemistry improved in a further seven patients. Treatment was well tolerated. Epirubicin given in this way is effective in patients with breast cancer and liver metastases. An initial deterioration in liver biochemistry may occur before there is a response to epirubicin.


*Br. J. Cancer* (1989), **60**, 938–941 © The Macmillan Press Ltd., 1989

### Weekly epirubicin for breast cancer with liver metastases and abnormal liver biochemistry

C.J. Twelves, S.M. O'Reilly, R.E. Coleman, M.A. Richards & R.D. Rubens

*Imperial Cancer Research Fund Clinical Oncology Unit, Guy's Hospital, London SE1 9RT, UK.*

**Summary** Thirty-six consecutive patients with breast cancer and liver metastases with abnormal liver biochemistry were treated with epirubicin 25 mg $m^{-2}$ i.v. weekly. No dose modification was made for abnormal liver biochemistry, but dose intensity was adjusted by delaying treatment according to myelosuppression. The UICC overall response rate according to UICC criteria was 11/36 (30%) and median response duration was 27 weeks. Liver biochemistry improved in a further seven patients. Treatment was well tolerated. Epirubicin given in this way is effective in patients with breast cancer and liver metastases. An initial deterioration in liver biochemistry may occur before there is a response to epirubicin.

Liver metastases are common in patients with advanced breast cancer. The prognosis is worse for these patients than after recurrence in either soft tissue (Fentiman *et al.*, 1986) or bone (Coleman & Rubens, 1987). Survival is particularly poor in patients with liver metastases and abnormal liver biochemistry (Swenerton *et al.*, 1979; Zinser *et al.*, 1987; S.M. O'Reilly, in preparation). The treatment of patients with liver metastases is difficult. Responses to endocrine therapy are uncommon (Australia & New Zealand Breast Cancer Trials Group, 1986). The administration of chemotherapy may be complicated by the liver's role in both cytotoxic drug activation, as for cyclophosphamide (Bagley *et al.*, 1973), and metabolism, as with doxorubicin (Benjamin *et al.*, 1974). Moreover, although anthracyclines are the most active single agents in patients with breast cancer (Steiner *et al.*, 1983), toxicity can be severe in patients with impaired liver function and reduced hepatic drug clearance (Benjamin *et al.*, 1974).

Epirubicin (4'-epidoxorubicin) is a new anthracycline, structurally related to doxorubicin and with similar activity in patients with advanced breast cancer (Brambilla *et al.*, 1986). It is eliminated more rapidly than doxorubicin (Camaggi *et al.*, 1988) and, at equimolar doses, is less toxic than doxorubicin (Brambilla *et al.*, 1986). We report our experience of the efficacy and toxicity of epirubicin in patients with breast cancer and liver metastases who have abnormal liver biochemistry.

Patients received a low dose of epirubicin on a weekly schedule, with the aim of giving a high dose intensity whilst minimising toxicity.

### Patients and methods

Thirty-six patients with histologically proven breast cancer, progressive liver metastases and abnormal liver biochemistry were entered into this phase II study. Eligible patients had liver metastases confirmed by technetium sulphur colloid radionuclide scan or ultrasound scan, and either a serum aspartate transaminase (AST) more than twice the upper limit of normal or an elevated serum bilirubin. Four patients had received previous adjuvant chemotherapy, three with a combination of cyclophosphamide, methotrexate and 5-fluorouracil (CMF) and one with L-PAM. A further four patients had undergone earlier chemotherapy with CMF for advanced breast cancer. Patients who had received previous anthracycline treatment were excluded from the study.

Epirubicin 25 mg $m^{-2}$ intravenously was given every 7 days. Liver biochemistry tests were repeated weekly but dose adjustments were not made for raised serum transaminases or bilirubin. However, chemotherapy was given only if the white blood cell count was above $2.0 \times 10^9 l^{-1}$ and platelet count above $70 \times 10^9 l^{-1}$. If the blood count fell below these levels, treatment was delayed until these counts were reached. With each course of chemotherapy patients received antiemetics, in most cases metoclopramide (20 mg i.v.) either alone or with dexamethasone (5–20 mg i.v.).

In these patients, the liver was the dominant site of disease. Objective responses were assessed therefore by UICC criteria for liver metastases (Hayward *et al.*, 1977). A complete remission (CR) was defined as disappearance of all evidence of hepatic metastases on repeat liver scan, clinical examination and serum biochemistry. A partial remission (PR) required at least a 50% reduction in hepatic metastases on liver scan and a 50% or greater decrease in palpable hepatomegaly below the costal margin. In an attempt to evaluate the importance of changes in liver biochemistry, we have defined patients with a greater than 50% reduction in abnormalities of serum bilirubin or AST, but stable disease on UICC criteria, as biochemical responders.

It was planned that all patients receive a minimum of six and a maximum of 12 cycles of weekly epirubicin. If there was disease progression after six cycles, chemotherapy was stopped. Patients with responding or stable disease according to UICC criteria continued weekly epirubicin for a further six cycles unless unacceptable toxicity or disease progression were documented during this period. Patients who were in remission after 12 cycles, generally stopped chemotherapy and were observed without treatment. These patients were eligible for further chemotherapy, including epirubicin, at relapse.

Treatment toxicity was graded by the WHO classification (Miller *et al.*, 1981). Survival was measured from the date of first epirubicin treatment to death, and progression-free interval from the date of first epirubicin treatment to the date of progression.

### Results

Patient characteristics on entry to the study are shown in Table I. All 36 patients were assessable for response to chemotherapy, with a median follow-up of 38 weeks. They received a median of eight initial weekly epirubicin treatments (range 1–15) and 15 patients completed 12 courses of chemotherapy. Fifteen patients had a raised bilirubin on entry and the median AST was four times greater than the upper limit of the laboratory's normal range. The serum alkaline phophatase was raised in all patients, but 24 patients also had bone metastases. Alkaline phosphatase was therefore not used to diagnose liver metastases or to monitor response to treatment.

One patient achieved a complete remission (CR) and 10 a partial remission (PR) on weekly epirubicin. The responders included four of the 12 patients who were jaundiced before

Correspondence: C.J. Twelves.
Received 21 February 1989; and in revised form 31 July 1989.

**Table I** Patient characteristics on entry to the study

| | |
|---|---|
| No. of patients | 36 |
| Median age | 54 years (range 26–79) |
| Histology | |
| Infiltrating ductal | 28 |
| Infiltrating lobular | 2 |
| Unknown | 6 |
| Receptor status at initial diagnosis | |
| ER unknown | 17 |
| positive | 15 (79% of known patients) |
| negative | 4 (21%) |
| PR unknown | 16 |
| positive | 10 (50%) |
| negative | 10 (50%) |
| Previous chemotherapy | 8 (4 as adjuvant) |
| Previous endocrine therapy | 24 |
| Menstrual status | |
| Premenopausal | 9 |
| 1–5 years post-menopausal | 5 |
| > 5 years post-menopausal | 22 |
| UICC performance status | |
| 0 | 5 patients |
| 1 | 10 |
| 2 | 15 |
| 3 | 6 |
| Sites of assessable extrahepatic metastases | |
| Bone | 23 |
| Lymphatic | 8 |
| Skin | 6 |
| Lung | 3 |
| Pleura | 3 |
| Breast | 4 |
| Clinical signs of liver metastases | |
| Jaundice | 12 |
| Ascites | 3 |
| Hepatomegaly | 29 |
| Median AST (normal < 34) | 151 (72–392) |
| Median bilirubin (normal < 17) | 19 (7–287) |
| Imaging technique | |
| Radionuclide scan | 19 |
| Ultrasound | 16 |
| CT | 1 |

chemotherapy. All 11 patients who achieved an objective response (UICC) had improvement of liver biochemistry. Seven other patients had a biochemical response, but either had stable disease on repeat liver scan (four patients) or the scan was not repeated (three patients). In two of these patients, liver biochemistry returned to normal during chemotherapy, but the metastases were unchanged on repeat liver scan.

The objective response rate for liver metastases was 11/36 (30%, with 95% confidence limits of 15–45%) according to UICC criteria, and 18/36 (50%, with 95% confidence limits of 33–67%) if patients with a biochemical response are included. Responses were also observed in soft tissue, bone and other visceral metastases. Only two patients in whom liver metastases failed to respond to epirubicin achieved a response at other metastatic sites of disease. No patient who responded in the liver showed progression of disease elsewhere. Median progression free interval in the 11 UICC responders was 27 weeks (range 8–40), and in the seven biochemical responders 22 weeks (range 7–25). All patients who responded according to UICC criteria, and the biochemical responders, had improvement of symptoms including reduction in nausea, anorexia, hepatic pain and/or improvement in performance status.

During the first month of treatment, serum bilirubin or AST rose by at least 25% in seven of the 18 responders. Six of these seven patients had a raised serum bilirubin before chemotherapy. As chemotherapy continued liver biochemistry subsequently improved, and by 6 weeks these patients could clearly be distinguished from those with progressive disease in whom there was a steady deterioration. Figure 1 shows the two patterns of serial liver biochemistry we observed in patients who achieved a UICC response.

**Figure 1** Serial AST ——— and bilirubin — — — in (**a**) a responding patient with steady improvement in liver biochemistry, (**b**) a patient with initial deterioration in liver biochemistry who later responded.

Eleven patients received further chemotherapy at relapse. Six of these patients were treated again with epirubicin and two achieved a second response. Four patients were treated with a combination of cyclophosphamide, methotrexate and 5-fluoruoracil and one with cyclophosphamide alone, but none responded.

Median survival for all patients was 16 weeks (range 1–54) (Figure 2). There were no deaths directly related to chemotherapy but two patients died without evidence of disease progression, one from gastrointestinal haemorrhage and one with a pulmonary embolism which was confirmed at post-mortem. Twelve patients died within 6 weeks of starting chemotherapy. Ten of these 12 patients had had a 4-fold or greater elevation in initial AST which strongly predicted for early death ($P = 0.025$, Fisher's exact test). Survival for the biochemical responders was similar to that for UICC responders ($P = 0.14$, log rank test), and was significantly better than for patients with progressive disease ($P = 0.03$, log rank test).

All patients were evaluable for toxicity. Treatment was

**Figure 2** Survival – all patients.

generally tolerated well. Despite being offered scalp cooling, all patients who received six or more courses of epirubicin developed WHO grade 2 or 3 alopecia. Gastrointestinal toxicity was mild, with grade 3 stomatitis in two patients and grade 4 vomiting in one patient. Myelosuppression delayed 34 of 263 weekly treatments (13%). Treatment delays occurred in four of the 15 patients with a raised bilirubin before starting chemotherapy and in six of 21 patients who initially had a normal bilirubin. There was no grade 4 haematological toxicity, and there were no episodes of septicaemia.

### Discussion

The prognosis for breast cancer patients with liver metastases is poor. Treatment is palliative and the aim must be to combine maximum efficacy with acceptable toxicity. Doxorubicin is the most active single agent in breast cancer (Steiner *et al.*, 1983) and is more effective at high ($70\,mg\,m^{-2}$) than low ($35\,mg\,m^{-2}$) doses (Carmo-Pereira *et al.*, 1987). However, because anthracyclines are metabolised by the liver, elimination is delayed in patients with liver dysfunction (Benjamin *et al.*, 1984). Unfortunately there is only a poor correlation between conventional liver biochemical tests and anthracycline metabolism (Preiss *et al.*, 1987) and dose adjustments based on these tests are empirical (Benjamin *et al.*, 1974). Epirubicin is also metabolised principally by the liver (Camaggi *et al.*, 1982), and the manufacturers recommend dose reduction in patients with moderate or severe liver impairment as defined by raised serum bilirubin or bromsulphthalein clearance. In this study such dose modifications were not made. We adjusted dose intensity according to myelosuppression; in this way any effect of decreased epirubicin clearance due to impaired liver function led to a delay in treatment and reduction in dose intensity.

Few studies have been directed specifically at breast cancer patients with liver metastases (Zinser *et al.*, 1987; O'Reilly *et al.*, 1989). The majority of reports of chemotherapy in such patients have been derived from larger studies of patients with metastatic disease in a varity of sites. A review of these trials by Kemeny (1983) showed a response rate for liver metastases of between 30 and 75%. These results are not, however, directly comparable with those in the current study. Details of disturbances in liver biochemistry were not given, although abnormalities of liver biochemistry confer a poor prognosis (Swenerton *et al.*, 1979; Zinser *et al.*, 1987; O'Reilly *et al.*, 1989). Indeed, many studies have specifically excluded patients with abnormal liver biochemistry although at least a third of breast cancer patients have a 2-fold or greater elevation in AST at the time of diagnosis of liver metastases (Zinser *et al.*, 1987; S.M. O'Reilly, in preparation). It is important to evaluate treatment regimens in this group of patients.

We have treated a clearly defined group of breast cancer patients with liver metastases and abnormal liver biochemistry using a single drug, epirubicin, given weekly. Our results show that in these patients epirubicin given in this way achieves a response rate of 30% according to UICC criteria. This rises to 50% if patients with a biochemical response are included, and these patients had a significantly better prognosis than patients with progressive disease, suggesting that improved liver biochemistry may be a useful additional measure of response to chemotherapy. Moreover, seven of the 25 patients (28%) whose liver biochemistry deteriorated during the first month of treatment subsequently achieved either a response according to UICC criteria or a biochemical response. This may simply reflect a continuing deterioration in liver biochemistry before treatment has time to take effect, or a hepatotoxic effect of epirubicin in patients with severely disturbed liver function. Transient abnormalities of liver biochemistry have also been reported in patients with breast cancer receiving an adriamycin, 5-fluorouracil and cyclophosphamide regimen (Larroquette *et al.*, 1986), and patients with hepatocellular carcinoma treated with mitoxantrone (Yoshida *et al.*, 1988). Whatever the mechanism of the initial deterioration in liver biochemistry, it is important to note that, although liver biochemistry may be a useful indicator of response to chemotherapy, it is not a reliable measure of progression during the first few weeks of treatment. Chemotherapy should be continued for at least 6 weeks before response is assessed, as an initial deterioration in liver biochemistry does not necessarily indicate disease progression.

We adjusted dose intensity of epirubicin according to the severity of myelosuppression alone, regardless of liver biochemistry, and found no difference in the number of treatment delays between patients with normal or raised bilirubin. A conventional dose reduction might have exposed some patients to suboptimal chemotherapy and others to unacceptable toxicity. Comparison with historical results from Guy's Breast Unit suggests that epirubicin given in this way is more active than either mitoxantrone (O'Reilly *et al.*, 1989) or other chemotherapy (predominantly adriamycin at 3-weekly intervals or a combination of cyclophosphamide, methotrexate and 5-fluorouracil) in this group of patients (S.M. O'Reilly, in preparation). However, median survival in this study was only 16 weeks and, although responders gained symptomatic benefit, it remains uncertain whether chemotherapy improves survival in patients with liver metastases. Nevertheless, it seems likely that in this study epirubicin delayed death in some patients.

We conclude that patients with breast cancer and liver metastases who have abnormal biochemistry can usefully be treated with weekly epirubicin $25\,mg\,m^{-2}$, adjusting dose intensity according to myelosuppression. This treatment is tolerated well. However, an initial deterioration in liver biochemistry may occur before there is a response to epirubicin.

### References

AUSTRALIAN & NEW ZEALAND BREAST CANCER TRIALS GROUP (1986). A randomised trial in post-menopausal patients with advanced breast cancer comparing endocrine and cytotoxic therapy given sequentially or in combination. *J. Clin. Oncol.*, **4**, 186.

BAGLEY, C.M., BOSTICK, F.W. & DE VITA, V.T. (1973). Clinical pharmacology of cyclophosphamide. *Cancer Res.*, **33**, 226.

BENJAMIN, R.S., WIERNIK, P.H., WESLEY, M. & BACHER, N.R. (1974). Adriamycin chemotherapy, efficacy, safety and pharmacologic basis of an intermittent single high dose schedule. *Cancer*, **33**, 19.

BRAMBILLA, C., ROSSI, A., BONFANTE, V. & 4 others (1986). Phase II study of doxorubicin versus epirubicin in advanced breast cancer. *Cancer Treat. Rep.*, **70**, 261.

CAMAGGI, C.M., STROCCHI, E., TAMASSIA, V. & 7 others (1982). Pharmacokinetic studies of 4'epidoxorubicin in cancer patients with normal and impaired renal function and with hepatic metastases. *Cancer Treat. Rep.*, **66**, 1819.

CAMAGGI, C.M., COMPARSI, R., STROCCHI, E., TESTONI, F., ANGELELLI, B. & PANUTTI, F. (1988). Epirubicin and doxorubicin comparative metabolism and pharmacokinetics. *Cancer Chemother. Pharmacol.*, **21**, 221.

CARMO-PEREIRA, J., COSTA, F.O., HENRIQUES, E. & 4 others (1987). A comparison of two doses of adriamycin in the primary chemotherapy of diesseminated breast carcinoma. *Br. J. Cancer*, **56**, 471.

CHLEBOWSKI, R.T., IRWIN, L.E., PUGH, R.P. & 4 others (1979). Survival of patients with metastatic breast cancer treated with either combination or sequential chemotherapy. *Cancer Res.*, **39**, 4503.

COLEMAN, R.E. & RUBENS, R.D. (1987). The clinical course of bone metastases from breast cancer. *Br. J. Cancer*, **55**, 61.

FENTIMAN, I.S., LAVELLE, M.A., CAPLAN, D., MILLER, N., MILLIS, R.R. & HAYWARD, J.L. (1986). The significance of supraclavicular fossa node recurrence after radical mastectomy. *Cancer*, **57**, 908.

HAYWARD, J.L., CARBONE, P.P., HEUSON, J.C., KUMAOKA, S., SEGALOFF, A. & RUBENS, R.D. (1977). Assessment of response to therapy in advanced breast cancer. *Eur. J. Cancer*, **13**, 89.

KEMENY, N. (1983). The systemic chemotherapy of hepatic metastases. *Semin. Oncol.*, **10**, 148.

KENNEALEY, G.T., BARTON, B., MITCHELL, M.S. *et al.* (1978). Combination chemotherapy for advanced breast cancer. *Cancer*, **42**, 27.

LARROQUETTE, C.A., HORTOBAGYI, G.N., BUZDAR, A.V. & HOLMES, F.A. (1986). Subclinical hepatic toxocity during combination chemotherapy for breast cancer. *JAMA*, **256**, 2988.

MILLER, A.B., HOOGSTRATEN, B., STAQUET, M. & WINKLER, A. (1981). Reporting results of cancer treatment. *Cancer*, **47**, 207.

O'REILLY, S.M., COLEMAN, R.E. & RUBENS, R.D. (1989). Phase II study of mitoxantrone for liver metastases from breast cancer. *Cancer Chemother. Ther.* (in the press).

PREISS, R., MATTHAIS, M., SOHR, R., BROCKMANN, B. & HILLER, H. (1987). Pharmacokinetics of adriamycin, adriamycinol and antipyrine in patients with moderate tumour involvement of the liver. *J. Cancer Res. Oncol.*, **113**, 593.

SMALLEY, R.V., CARPENTER, J., BARTOLUCCI, A., VOGEL, C. & KRAUSS, S. (1977). A comparison of cyclophosphamide, adriamycin and 5-fluorouracil (CAF) and cyclophosphamide, methotrexate, 5-fluorouracil, vincristine, prednisolone (CMFVP) in patients with metastatic breast cancer. *Cancer*, **40**, 625.

STEINER, R., STEWART, J.F., CANTWELL, B.M.J., MINTON, M.J., KNIGHT, R.K. & RUBENS, R.D. (1983). Adriamycin alone or combined with vincristine in the treatment of advanced breast cancer. *Eur. J. Clin. Oncol.*, **19**, 1553.

YOSHIDA, T., OKAZAKI, N., YOSHINO, M., OHKURA, H., MIYAMOTO, K. & SHIMADO, Y. (1988). Phase II trial of Mitoxantrone in patients with hepatocellular carcinoma. *Eur. J. Clin. Oncol.*, **24**, 1897.

ZINSER, J.W., HORTOBAGYI, G.M., BUZDAR, A.U., SMITH, T.L. & FRASCHINI, G. (1987). Clinical course of breast cancer patients with liver metastases. *J. Clin. Oncol.*, **5**, 773.